# A Long-Term Study of a Lipid-Buprenorphine Implant in Rats

**DOI:** 10.1155/2018/2616152

**Published:** 2018-07-09

**Authors:** Michael Guarnieri, Cory Brayton, Betty M. Tyler

**Affiliations:** ^1^Johns Hopkins School of Medicine, Department of Neurological Surgery, Baltimore, MD, USA; ^2^Johns Hopkins School of Medicine, Department of Molecular and Comparative Pathobiology, Baltimore, MD, USA

## Abstract

Animal models to study opiates are of growing interest. We have examined the short-term safety of buprenorphine implants in Fischer F344/NTac rats treated with excess doses of a cholesterol-triglyceride suspension of buprenorphine. A single injection of 0.65 mg/kg afforded clinically significant blood levels of analgesia for 3 days. Chemistry, hematology, coagulation, and urinalysis values with 2- to 10-fold excess doses of the drug-lipid suspension were within normal limits. Histopathology findings were unremarkable. The skin and underlying tissue surrounding the drug injection were unremarkable. Here we report the results of a long-term follow-up study of female rats injected with 0.65 and 1.3 mg/kg. The 14-month evaluation showed no abnormal findings that could be attributed to the drug or lipid suspension. These results confirm the safety of cholesterol-triglyceride carrier systems for subcutaneous drug delivery in laboratory animals and suggest that this model may be used to study long-term effects of opiate therapy.

## 1. Introduction

Models of long-acting drug therapy in laboratory animals have been managed by adding drugs to the feed or water supplies. [1, 2] The utility of this strategy decreases when the food or water mixture may be released inadvertently to the environment or the drug is highly regulated, such as controlled substance. Alternative approaches have focused on long-acting drug implants made by combining a drug with biodegradable implants composed of lipids or polymers [3].

Polymers have been studied as dug carriers for neurooncology [4]. Side effects generally have been modest and localized when the polymer is implanted into neural tissue [5]. Less is known about biodegradable polymers for subcutaneous (SC) delivery of chemotherapy. Moderate to severe inflammatory reactions have been reported for SC implants of polymer-opiate constructs [6–11]. Fewer adverse events have been reported with lipid drug carriers [12]. Kent described an implantable cholesterol matrix that delivered large molecules such as insulin and growth hormone [13]. Grant and coworkers demonstrated that a phospholipid-morphine liposome had prolonged activity and greater safety in mice than the free drug [14]. Lipid-based carrier strategies have been refined for the delivery of several opiates [15]. Pontani and Misra described a cholesterol-triglyceride matrix for the long-term delivery of drugs to treat chronic pain and opiate addiction [16]. Cholesterol-triglyceride vehicles appeared to provide a promising carrier to examine the delivery of antibiotics, anti-inflammatory drugs, and analgesics for pain management in animals. Yet, solid cholesterol-based implants can be sequestered by interstitial tissues and create drug-containing depots with uncertain release kinetics [17]. Subsequent studies have demonstrated that cholesterol suspensions provide consistent release kinetics with little evidence of tissue sequestration effects [18].

We examined the safety of cholesterol-triglyceride suspensions of buprenorphine in mice and rats using US Food and Drug Administration (FDA) Target Animal Safety (TAS) drug-development protocols. Histopathology examinations were performed on mice and rats treated with up to tenfold excess of the intended dose of drug and control animals following 4- and 12-day drug trials [19–21]. The examinations, including studies of the SC area of drug implantation, were unremarkable. There were no significant differences between the drug-treated and control animals. To examine the long-term safety of the lipid suspension, we dosed groups of female rats with the intended dose (0.65 mg/kg), 1.3 mg/kg of drug, and control, drug-free matrix. The rats were maintained for 14 months and euthanized to provide long-term clinical pathology data. As shown in the following report, there were no significant differences between the weights, laboratory chemistry, hematology, and necroscopy reports in drug-treated and control rats.

## 2. Methods

### 2.1. Animals and Husbandry

Studies were approved by a Johns Hopkins University Institutional Animal Care and Use Committee (IACUC). The IACUC protocol complies with the National Research Council's Guide for the Care and Use of Laboratory Animals and fulfills the requirements of the Association for the Assessment and Accreditation of Laboratory Animal Care, International Program. Fischer F344 (F344/NTac) rats, 6-8-week-old, female, 120-130 g, were obtained from Taconic Farms (Hudson NY) and housed in an environmentally controlled room which maintained the temperatures of 68 to 79°F. Monthly health surveillance was conducted by a soiled-bedding sentinel system. Sentinel rats were negative for pneumonia virus of mice, reovirus, Sendai virus, lymphocytic choriomeningitis virus, rat coronavirus, sialodacryoadenitis virus, rat parvovirus, Kilham rat virus, Toolan H1 parvovirus, rat theilovirus, cilia-associated respiratory bacillus,* Pneumocystis carinii, Mycoplasma pulmonis*, and pinworms throughout the study.

The facility maintained a relative humidity of 30 to 70% with a 12-hour light/12-hour dark cycle from 6 AM to 6 PM. Animals were group housed (up to 3 per cage) during the quarantine and acclimation period based on group/sex designation. The animals were quarantined and acclimated for six days prior to dosing. No disease-related signs were noted during the quarantine/acclimation period. Prior to being placed on test, a Clinical Veterinarian approved the animals for study use. All animals appeared normal prior to dosing. After the drug injection, rats were housed 1 per cage for 7 days to prevent redosing by coprophagy. On day 8, rats were housed 2 per cage. Housing consisted of soft fiber contact bedding (Carefresh Natural bedding; Ferndale WA) ad libitum access to Harlan TEKLAD Certified Global Rodent Diet 2016C (Harlan TEKLAD, Indianapolis IN) and ad libitum access to drinking water (Baltimore City Water System, Baltimore MD) in disposable water bottles. The animals were provided ad libitum access to Harlan TEKLAD Certified Global Rodent Diet 2016C (Harlan TEKLAD, Indianapolis IN). Rats were provided enrichment devices of polycarbonate red tubes (Bio Services, Uden, NL). Their static microisolator cages were changed weekly.

### 2.2. Experimental Design

The label dose of 0.65 mg/kg of buprenorphine, which provides 2-3 days of clinically significant blood levels of drug, was established in bioequivalence trials and efficacy studies using male and female rats [21]. The 12 female rats in this study were divided into groups of 4 rats provided 0.0, 0.65, and 1.3 mg/kg buprenorphine. Cage side evaluations were conducted twice daily in the morning and afternoons for two weeks by a veterinarian who was blind to the dose group. Rats were evaluated at weekly intervals by staff veterinary services thereafter. Rats were scheduled for harvesting and clinical pathology studies at 1 year and actual 14 months. Anomalies or abnormalities discovered during necroscopy were further examined by histopathology. The hypothesis was that rats treated with the highest dose would have tissue changes near the injection site not found in the control group.

### 2.3. Drug Delivery

The buprenorphine-free control suspension consisted of cholesterol and glycerol tristearate (96:4) suspended in medium-chain triglyceride oil (8 mg/100 uL). The drug suspension consisted of buprenorphine, cholesterol, glycerol tristearate, suspended in medium-chain triglyceride (MCT) oil, and Miglyol 812, (Sasol, Hamburg Germany), 8 mg/100 uL, trade name Animalgesics for Mice. Control and drug suspensions were supplied by Animalgesics Labs (Millersville MD). To limit stress associated with constraining conscious animals for SC injections, rats were anesthetized with an intraperitoneal (IP) solution of ketamine, 80 mg/kg, and xylazine, 8 mg/kg, in a saline solution containing 14.25% ethyl alcohol. Each rat was injected with the designated dose of test article or buprenorphine-free control suspension before they recovered from anesthesia. The dose was administered SC on the mid-dorsal area about 1 cm rostral to the surgical incision using a 21 gauge needle (BD, Franklin NJ) attached to a 1 mL BD Tuberculin syringe. Following dose administration, animals were transferred to a clean cage on a heating pad until recovered. Once the animal regained consciousness and demonstrated normal movement, and with the absence of signs of distress, it was returned to its home cage. Posttreatment distress was not observed.

### 2.4. Clinical and Anatomic Pathology

At the 14 months' time point, euthanasia was administered by CO_2_ inhalation and blood collection by and exsanguination cardiocentesis, followed by a thoracotomy.

Hematology examination (CBC) included red blood cell (RBC) count, hemoglobin, hematocrit, mean corpuscular volume, mean corpuscular hemoglobin, mean corpuscular hemoglobin concentration, platelet count, white blood cell (WBC) count, differential blood cell count, and blood smear. Serum chemistry tests included glucose, urea nitrogen, creatinine, total protein, albumin, globulin (as calculation), total cholesterol, alanine aminotransferase, alkaline phosphatase, aspartate aminotransferase, calcium, sodium, potassium, chloride, and phosphate.

Necropsy was then performed for each animal. Tissues for histopathology were immersion fixed in individually labeled containers containing 10% neutral-buffered formalin. The transfer was performed and documented by the histology lab. Containers were labeled with study number, date, group number, and animal number.

Organ weights included adrenals, brain, heart, kidneys, liver, ovaries, spleen, and uterus with cervix. Histopathology was assessed on any gross lesions, dorsal skin surrounding the injection site as previously described, and esophagus, heart, kidneys, liver, lung, lymph nodes, and spleen.

### 2.5. Statistics

Statistical analyses (mean, standard deviations, N) were conducted for organ weight and clinical pathology data comparing treated groups to the control group. Because of the lack of variation in the data (see the Results) further analyses including one-way Analysis of Variance (ANOVA) and Dunnett's t-test (1955, 1964) were not used.

## 3. Results

All rats survived to the scheduled termination date. Signs of excessive grooming and self-gnawing were noted in one rat in the 1.3 mg/kg dose group at days 1-2 posttreatment. The observer noted the findings as a comment on the animal's chart. There was no evidence of an open wound. Since day 3, there were no remarkable changes in animal behavior.

The outcome weight of the three groups was similar: 265±23 g, 255±30 g, and 257±19 g for the rats in the 0.0, 0.65, and 1.3 mg/kg dose groups, respectively. There was no significant difference in organ weights of liver, spleen, heart, kidneys (both), brain, adrenal glands (both), uterus + cervix, and ovaries (both).

There was no significant difference in the hematology values between the three groups. Average group differences were noted between the two clinical chemistry values. Blood urea nitrogen (BUN) values were 16.50 ± 1.29, 15.50 ± 1.00, and 13.25 ±0.96 mg/dL for the control, 0.65 and 1.3 mg/kg groups, respectively. Alkaline phosphatase (ALP) values were 177.75 ± 34.45, 146.00 ± 22.70, and 109.00 ±21.52 (U/L), respectively.

On gross examination, 2 rats in the vehicle control group and 1 in the 1.3 mg/kg had mild dorsal neck/interscapular skin excoriations or mild crusting. Injection sites could not be distinguished from surrounding skin in other rats. On microscopic examination one rat in the control group and one rat in the 0.65 mg/kg dose group had mild ulceration and chronic dermatitis involving the dorsal neck/interscapular skin, but mild-moderate hemorrhage and serosuppurative crust that were consistent with more recent injury, possibly related to conspecific trauma such as aggressive neck grooming by cage mates. Other findings included one rat in the control group: cystic uterine (endometrial) polyp less than 4mm diameter, and one rat in the 0.65 mg/kg dose group: ovarian cyst ~1cm diameter. Mild cardiac changes (inflammation, degeneration, and fibrosis), mild nephropathy with pigment in tubule epithelium, mild lung inflammatory changes, and micromineralization of lung vasculature were observed in all groups. Pigmented macrophages in lymph nodes and spleen were observed in all rats. One rat in the 0.65 mg/kg group had an ovarian cyst and one in the vehicle control group had a uterine polyp. Pigment at various sites was consistent with hemosiderin, hematoidin, and or ceroid/lipofuscin. No unique microscopic lesions were associated with the test article.

## 4. Discussion

The objective of this study was to evaluate the long-term safety of a lipid suspension of buprenorphine for delivery of an opiate analgesic in female F344 rats. Female rats were chosen for a long-term trial because female rats may have increased susceptibility to opiates [22], although sex differences have not been fully determined. The results confirmed that lipid-based delivery system caused no significant adverse effects in a F344 rat model. One rat illustrated signs of nausea at days 1-2, signs that have been observed previously [23]. The incidence of nausea in this model appears similar to the observed incidence of nausea in human patients treated with opioid analgesia [24].

There were no remarkable differences in hematology parameters between control and drug-treated rats. Clinical chemistry values for serum BUN and ALK in the rats given 1.3 mg/kg dose were decreased compared to control values and in rats given the intended dose of 0.65 mg/kg. The clinical significance of these differences remains uncertain.

Weight loss has been cited as a deterrent to the use of postsurgical buprenorphine analgesia, and it has been linked to significant morbidity secondary to gastrointestinal blockage associated with hardwood bedding [25, 26]. A number of reports between 2000 and 2010 described weight loss in rats treated with buprenorphine without reference to the bedding used in the experiment [27, 28], or they report using hardwood bedding without reference to previous reports associating hardwood bedding with pica [27]. Previous studies have demonstrated that the risk of pica-related gastric distress can be controlled by the appropriate choice of bedding [29]. There were no significant differences between the organ weights or appearances of the drug-treated rats. Body weights also were similar. The studies reported here confirm this observation.

The results of this study are compromised by the failure to include male rats and use of higher doses of drug. It did not include a saline-control group to establish the safety of the lipid suspension.

## 5. Conclusion

There do not appear to be clinically significant treatment-related effects following a subcutaneous injection of an extended release lipid suspension of buprenorphine at 0.65 and 1.3 mg/kg dose. Although several clinical pathology findings exceeded normal limits, there were no correlated changes or findings in body weights, clinical observations, organ weights, or microscopic evaluation of tissues.

## Data Availability

The data used to support the findings of this study are available from the corresponding author upon request.

## References

[1] Abelson K. S. P., Jacobsen K. R., Sundbom R., Kalliokoski O., Hau J. (2012). Voluntary ingestion of nut paste for administration of buprenorphine in rats and mice. *Laboratory Animals*.

[2] Molina-Cimadevila M. J., Segura S., Merino C., Ruiz-Reig N., Andrés B., de Madaria E. (2014). Oral self-administration of buprenorphine in the diet for analgesia in mice. *Laboratory Animals*.

[3] Guo D., Huang J. (2017). New developments in long-acting injectable nanoformulations. *Global Journal of Pharmacy and Pharmaceutical Sciences*.

[4] Tyler B., Wadsworth S., Recinos V. (2011). Local delivery of rapamycin: a toxicity and efficacy study in an experimental malignant glioma model in rats. *Neuro-Oncology*.

[5] Tamargo R. J., Epstein J. I., Reinhard C. S., Chasin M., Brem H. (1989). Brain biocompatibility of a biodegradable, controlled-release polymer in rats. *Journal of Biomedical Materials Research Part B: Applied Biomaterials*.

[6] Clark T. S., Clark D. D., Hoyt R. F. (2014). Pharmacokinetic comparison of sustained-release and standard buprenorphine in mice. *Journal of the American Association for Laboratory Animal Science*.

[7] Carbone E. T., Lindstrom K. E., Diep S., Carbone L. (2012). Duration of action of sustained-release buprenorphine in 2 strains of mice. *Journal of the American Association for Laboratory Animal Science*.

[8] Foley P. L., Liang H., Crichlow A. R. (2011). Evaluation of a sustained-release formulation of buprenorphine for analgesia in rats. *Journal of the American Association for Laboratory Animal Science *.

[9] Divincenti L., Meirelles L. A. D., Westcott R. A. (2016). Safety and clinical effectiveness of a compounded sustained-release formulation of buprenorphine for postoperative analgesia in New Zealand white rabbits. *Journal of the American Veterinary Medical Association*.

[10] Nunamaker E. A., Stolarik D. F., Ma J., Wilsey A. S., Jenkins G. J., Medina C. L. (2014). Clinical efficacy of sustained-release buprenorphine with meloxicam for postoperative analgesia in Beagle dogs undergoing ovariohysterectomy. *Journal of the American Association for Laboratory Animal Science *.

[11] Nunamaker E. A., Halliday L. C., Moody D. E., Fang W. B., Lindeblad M., Fortman J. D. (2013). Pharmacokinetics of 2 formulations of buprenorphine in macaques (macaca mulatta and macaca fascicularis). *Journal of the American Association for Laboratory Animal Science *.

[12] Mishra D. K., Dhote V., Bhatnagar P., Mishra P. K. (2012). Engineering solid lipid nanoparticles for improved drug delivery: promises and challenges of translational research. *Drug Delivery and Translational Research*.

[13] Kent J. S. (June 1984). *Cholesterol matrix delivery system for sustained release of macromolecules*.

[14] Grant G. J., Vermeulen K., Zakowski M. I., Stenner M., Turndorf H., Langerman L. (1994). Prolonged analgesia and decreased toxicity with liposomal morphine in a mouse model. *Anesthesia & Analgesia*.

[15] Smith L. J., Kukanich B. K., Krugner-Higby L. A., Schmidt B. H., Heath T. D. (2013). Pharmacokinetics of ammonium sulfate gradient loaded liposome-encapsulated oxymorphone and hydromorphone in healthy dogs. *Veterinary Anaesthesia and Analgesia*.

[16] Pontani R. B., Misra A. L. (1983). A long-acting buprenorphine delivery system. *Pharmacology Biochemistry and Behavior*.

[17] Guarnieri M., Tyler B., Detolla L., Zhao M., Kobrin B. (2014). Subcutaneous implants for long-acting drug therapy in laboratory animals may generate unintended drug reservoirs. *Journal of Pharmacy and Bioallied Sciences*.

[18] Schildhaus N., Trink E., Polson C. (2014). Thermal latency studies in opiate-treated mice. *Journal of Pharmacy and Bioallied Sciences*.

[19] DeTolla L., Sanchez R., Khan E., Tyler B., Guarnieri M. (2014). Subcutaneous Implants of Buprenorphine-Cholesterol-Triglyceride Powder in Mice. *Journal of Veterinary Medicine*.

[20] Traul K. A., Romero J. B., Brayton C. (2014). Safety studies of post-surgical buprenorphine therapy for mice. *Laboratory Animals*.

[21] Guarnieri M., Brayton C., Sarabia-Estrada R., Tyler B., McKnight P., DeTolla L. (2017). Subcutaneous Implants of a Cholesterol-Triglyceride-Buprenorphine Suspension in Rats. *Journal of Veterinary Medicine*.

[22] Bartok R. E., Craft R. M. (1997). Sex differences in opioid antinociception. *Journal of Pharmacology and Experimental Therapeutics*.

[23] Cowan A., Sarabia-Estrada R., Wilkerson G., McKnight P., Guarnieri M. (2016). Unanticipated Adverse Events Associated with an Extended-Release Buprenorphine Toxicity Study in Fischer 344 Rats. *Laboratory Animals*.

[24] Aparasu R., McCoy R. A., Weber C., Mair D., Parasuraman T. V. (1999). Opioid-induced emesis among hospitalized nonsurgical patients: effects on pain and quality of life. *Journal of Pain and Symptom Management*.

[25] Clark J.A. J., Myers P. H., Goelz M. F., Thigpen J. E., Forsythe D. B. (1997). Pica behavior associated with buprenorphine administration in the rat. *Laboratory Animals*.

[26] Bender H. M. (1998). Pica behavior associated with buprenorphine administration in the rat. *Laboratory Animal Science*.

[27] Jacobson C. (2000). Adverse effects on growth rates in rats caused by buprenorphine administration. *Laboratory Animals*.

[28] Brennan M. P., Sinusas A. J., Horvath T. L., Collins J. G., Harding M. J. (2009). Correlation between body weight changes and postoperative pain in rats treated with meloxicam or buprenorphine. *Laboratory Animals*.

[29] Jablonski P., Howden B. O., Baxter K. (2001). Influence of buprenorphine analgesia on post-operative recovery in two strains of rats. *Laboratory Animals*.

